# Platelet-Type Phosphofructokinase Drives Immune Checkpoint Coexpression and Metabolic Pathway Activation in Liver Cancer: The Cancer Genome Atlas–Based and Experimental Validation

**DOI:** 10.14309/ctg.0000000000001041

**Published:** 2026-04-27

**Authors:** Lilan Cen, Zhe Tian, Haoming Hua, Shengyi Li, Wanling Chen, Junhua Lu

**Affiliations:** 1 Department of Infectious Disease, Guangxi Academy of Medical Sciences, The People's Hospital of Guangxi Zhuang Autonomous Region, Nanning, China;; 2 Department of Pulmonary and Critical Care Medicine, Life Science and Clinical Medicine Research Center, Affiliated Hospital of Youjiang Medical University for Nationalities, Baise, China;; 3 Department of Pathology, The Second People's Hospital of Bengbu, Bengbu, China;; 4 Department of Infection, Baise People's Hospital, Baise, China.

**Keywords:** hepatocellular carcinoma, PFKP, glycolysis, tumor microenvironment, immunotherapy

## Abstract

**INTRODUCTION:**

Hepatocellular carcinoma (HCC) is associated with poor clinical outcomes, underscoring the need to identify novel biomarkers and therapeutic targets. Platelet-type phosphofructokinase (PFKP), a key rate-limiting enzyme in glycolysis, is involved in tumor metabolic reprogramming, but its role in HCC prognosis, immune regulation, and therapeutic responsiveness remains incompletely understood.

**METHODS:**

We combined transcriptomic profiles and clinical information from The Cancer Genome Atlas with analyses of clinical HCC specimens. PFKP expression, survival associations, functional enrichment, and tumor immune microenvironment characteristics were systematically evaluated. Drug sensitivity was predicted using data from the Genomics of Drug Sensitivity in Cancer database. Validation was performed by immunohistochemistry in patient samples and by Western blotting in PFKP-overexpressing HepG2 cells treated with or without the glycolysis inhibitor 2-deoxy-d-glucose.

**RESULTS:**

PFKP expression was markedly increased in HCC and was independently associated with unfavorable survival. Elevated PFKP levels were linked to an immunoregulatory microenvironment and increased expression of immune checkpoint molecules (PD-L1, CTLA-4), which was confirmed by immunohistochemistry. High PFKP expression was also associated with predicted greater responsiveness to immune checkpoint blockade and distinct drug sensitivity profiles. Mechanistically, PFKP overexpression increased the expression of key glycolytic enzymes, enhanced PD-L1 expression, and was accompanied by activation of mechanistic target of rapamycin (mTOR)-related signaling, whereas these effects were attenuated by glycolytic inhibition.

**DISCUSSION:**

PFKP is a critical link between glycolysis and immune suppression in HCC. It promotes immune checkpoint expression and mTOR activation, suggesting a role in immune evasion. PFKP represents a dual biomarker for prognosis and immunotherapy response, and a promising target for combined metabolic and immune-based therapies.

## Graphical abstract

**Figure:**
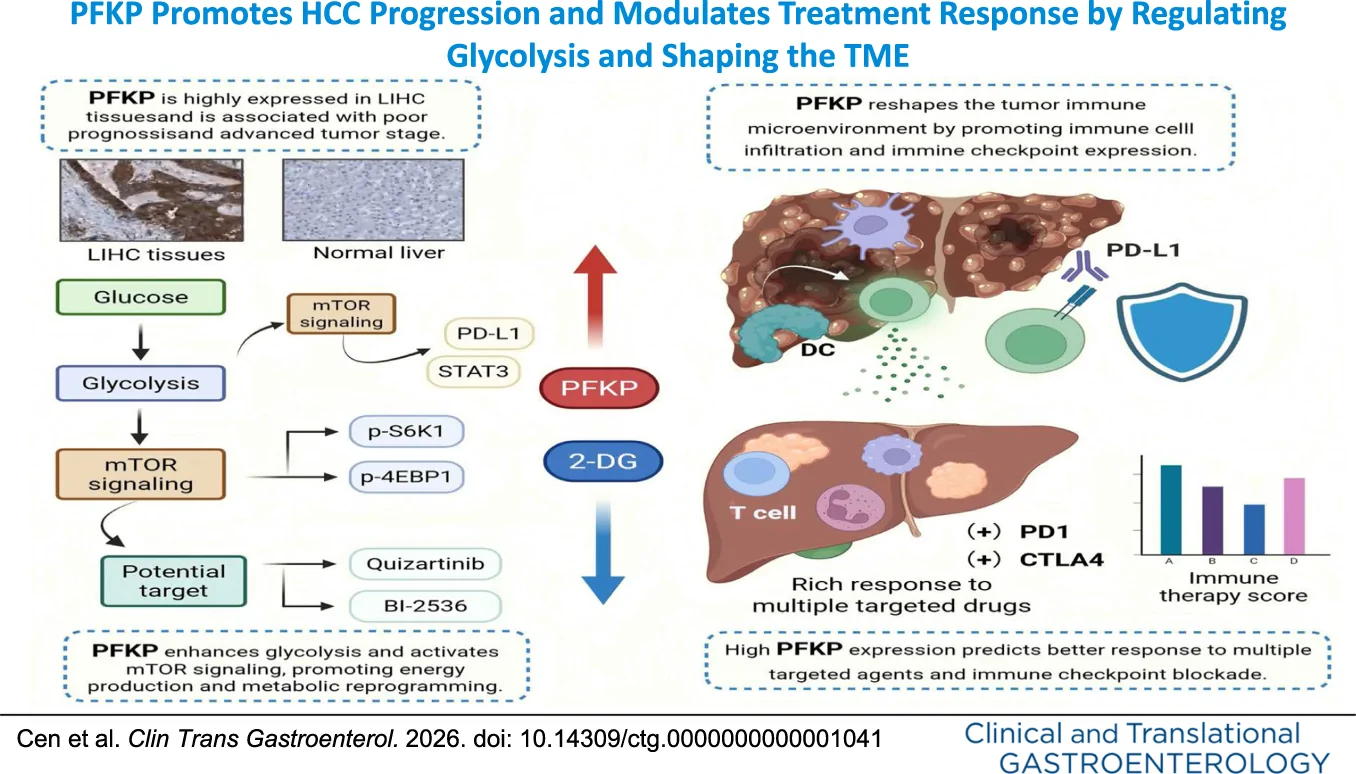

## INTRODUCTION

Hepatocellular carcinoma (HCC) is among the most prevalent malignant tumors worldwide and remains a major cause of cancer-associated death, posing a major threat to human health (1). Because HCC is often diagnosed at an advanced stage and conventional treatments, including surgical resection, radiotherapy, chemotherapy, and targeted therapy, provide limited benefit in many patients, overall clinical outcomes remain poor (2,3). In recent years, immune checkpoint inhibitors (ICIs) have become an important component of systemic therapy for HCC, particularly for patients with unresectable or metastatic disease, although their overall efficacy is still modest (4,5). However, many patients experience primary or acquired resistance, transient responses, or immune evasion during ICI treatment, and substantial interpatient heterogeneity continues to restrict the broader clinical benefit of immunotherapy (6). Therefore, identifying novel molecular biomarkers to support risk stratification, predict immunotherapeutic responses, and inform combination treatment strategies has emerged as a key research priority.

Aberrant glycolysis is a hallmark of tumor metabolic reprogramming and is commonly described as the “Warburg effect” (7,8). Compared with normal cells, cancer cells depend more heavily on aerobic glycolysis to support rapid proliferation by generating both energy and biosynthetic intermediates (9). Beyond supporting tumor growth, enhanced glycolysis reshapes the tumor microenvironment (TME) through lactate accumulation, extracellular acidification, and induction of immunosuppressive factors, thereby contributing to immune evasion (10). Increasing evidence suggests that glycolytic reprogramming can impair CD8^+^ T-cell function, promote the infiltration of regulatory T cells and myeloid-derived suppressor cells, and upregulate immune checkpoint molecules such as PD-L1, collectively influencing antitumor immune responses (11,12). Therefore, clarifying the crosstalk between glycolytic pathways and immune regulation, especially by identifying key regulators with both metabolic and immunomodulatory roles, may provide valuable insight for improving immunotherapy in HCC.

Platelet-type phosphofructokinase (PFKP) is a major rate-limiting enzyme in the glycolytic pathway that catalyzes the phosphorylation of fructose-6-phosphate to fructose-1,6-bisphosphate, thereby regulating glycolytic flux (13,14). Previous studies have shown that PFKP is upregulated in multiple malignancies, including breast cancer, lung cancer, and glioma and is closely linked to tumor progression, invasive behavior, and unfavorable prognosis (15,16). Emerging evidence also suggests that PFKP may influence pathways such as PI3K/AKT/mechanistic target of rapamycin (mTOR), thereby affecting the expression of immunoregulatory factors and remodeling the TME (17). However, despite the recognized importance of metabolic reprogramming in cancer (18,19) and the evolving systemic treatment landscape for HCC (20,21), the specific metabolic determinants that contribute to immune evasion in HCC remain insufficiently understood. In particular, the expression pattern and clinical relevance of PFKP in HCC, especially its associations with immune checkpoints, immune infiltration, and tumor microenvironmental features, have not been fully clarified. It also remains unclear whether PFKP primarily supports metabolic demands or may additionally participate in shaping the immunoregulatory state of HCC (22). Given the close interplay between metabolic dysregulation and immune suppression in HCC, PFKP may represent a candidate link between glycolytic metabolism and immune regulation, warranting further investigation of its prognostic and therapeutic relevance.

This study aimed to characterize the expression pattern of PFKP in HCC and evaluate its clinical relevance, with a focus on its potential role as a dual-function metabolic-immune regulatory factor. We systematically assessed the associations between PFKP and patient prognosis, immune checkpoint expression, TME status, and therapeutic sensitivity, to elucidate whether PFKP contributes to immune evasion in HCC through the glycolysis-mTOR signaling axis and whether it is involved in shaping the response to immunotherapy. Our findings may support the potential value of PFKP as a candidate biomarker for prognosis prediction and therapeutic stratification in HCC, while also providing insight into the rationale for combined metabolic-immune targeting strategies. Collectively, this study expands the current understanding of metabolic-immune crosstalk in HCC and may offer a basis for future personalized therapeutic development.

## METHODS

### Clinical sample collection and ethics

HCC specimens were collected from patients who underwent radical resection at our hospital between 2022 and 2023, including 5 tumor tissues and 5 matched adjacent normal liver tissues. Primary HCC was confirmed by both pathological and radiological examination. Patients with concurrent malignancies, severe extrahepatic diseases, or a history of radiotherapy or chemotherapy within 6 months before surgery were excluded. All samples were collected with a cold ischemia time of no more than 30 minutes, fixed in 4% neutral formalin for 24 hours, and processed into formalin-fixed paraffin-embedded tissue blocks. The samples were stored at room temperature in a dry, light-protected environment. All tissue sections were independently reviewed in a blinded manner by 2 senior pathologists, and any discrepancies were resolved by discussion or third-party adjudication. This study was approved by the Institutional Ethics Committee of our institution (Approval No. 20240306; approved on March 8, 2024), and written informed consent was obtained from all patients. Clinical and pathological information was deidentified before analysis. Samples from our institution were used as an independent histological and immunohistochemistry (IHC) validation cohort, separate from the The Cancer Genome Atlas (TCGA) discovery dataset.

### Data acquisition and preprocessing

RNA sequencing data and corresponding clinical information for the TCGA-LIHC cohort were downloaded from the Genomic Data Commons (GDC) Data Portal (https://portal.gdc.cancer.gov/) in December 2023. Both HTSeq-Counts and HTSeq-fragments per kilobase of transcript per million mapped reads (FPKM) data generated on the basis of the GRCh38 reference genome were obtained. Only samples annotated as “Primary Tumor” or “Solid Tissue Normal” were included. When multiple sequencing aliquots were available for the same patient, only the first eligible sample was retained. Samples lacking complete clinical information or follow-up data were excluded. Ultimately, 374 tumor samples and 50 adjacent normal samples were included for descriptive analysis. Data were log2-transformed and normalized using R software (version 4.2.2).

### Expression pattern analysis of PFKP in pan-cancer and HCC

RNA sequencing data (HTSeq-Counts and FPKM) from 33 cancer types and matched normal tissues were retrieved from the TCGA Pan-Cancer Atlas and integrated through the University of California, Santa Cruz Xena platform based on the GRCh38 reference genome. FPKM values were converted to transcripts per million (TPM), followed by log_2_(TPM+1) transformation for descriptive analysis. Intersample batch effects were removed with the ComBat algorithm implemented in the sva R package. Differences in PFKP expression among cancer types were evaluated using the Mann–Whitney *U* test, whereas paired comparisons between HCC tumors and adjacent nontumor tissues were performed with the Wilcoxon signed-rank test (R package: stats). All analyses were 2-sided, and *P* < 0.05 was considered statistically significant.

### Correlation analysis of PFKP-associated genes and chord diagram construction

In the HCC cohort (n = 374), correlations between PFKP expression and all other transcripts were calculated using the log_2_(TPM + 1) expression matrix and Pearson correlation analysis. Genes meeting the thresholds of |r| > 0.6 and false discovery rate (FDR) < 0.05 were considered significantly correlated, with *P* values adjusted using the Benjamini–Hochberg method (R package: psych, version 2.2.9). The top 10 genes showing the strongest correlations with PFKP were selected, and their relationships were visualized using a chord diagram generated with the circlize R package (version 0.4.16).

### Differential expression and gene ontology/Kyoto Encyclopedia of Genes and Genomes functional enrichment analysis

The TCGA-liver hepatocellular carcinoma (LIHC) cohort was stratified into high-PFKP and low-PFKP expression groups (n = 187 each) according to the median value of PFKP expression. Differential expression analysis was conducted on raw HTSeq-Counts data using the DESeq2 R package (version 1.38.0). Genes meeting the criteria of |log_2_ fold change| > 1 and FDR < 0.05 after Benjamini–Hochberg correction were considered differentially expressed. The identified differentially expressed genes were further analyzed for gene ontology (GO) biological process (BP) and Kyoto Encyclopedia of Genes and Genomes (KEGG) pathway enrichment analyses using the ClusterProfiler (version 4.2.2) and org.Hs.eg.db (version 3.14.0) R packages. Entrez Gene IDs were used for annotation, and all genes detected in the TCGA-LIHC dataset were defined as the background set. Enrichment results with *P* < 0.05 and *q* < 0.05 were considered significant. Volcano plots and heatmaps were generated using ggplot2 (version 3.3.6) and pheatmap (version 1.0.12), respectively.

### Gene set enrichment analysis enrichment analysis

Gene set enrichment analysis (GSEA) was conducted with the Broad Institute GSEA software (version 4.3.2). Gene set files were downloaded from MSigDB version 7.5.1, including c2.cp.kegg.v7.5.1.symbols.gmt (KEGG canonical pathways) and c5.go.bp.v7.5.1.symbols.gmt (GO BP). The analysis parameters were set as follows: permutation type = phenotype, number of permutations = 1,000, collapse dataset to gene symbols = false, and gene set size ranging from 15 to 500. Pathways with |NES| > 1, nominal *P* < 0.05 and FDR *q* < 0.25 were considered significantly enriched. Enrichment plots were generated using the built-in GSEA modules and further visualized in R (version 4.2.2) with the ggplot2 package (version 3.3.6), including bar plots, enrichment curves, and ridge plots.

### ESTIMATE immune scoring analysis

TME profiling of HCC samples was performed using the estimate R package (version 1.0.13). Log_2_(TPM + 1)-transformed expression matrices were used as input to calculate Immune Scores and Stromal Scores. According to the median PFKP expression level, samples were assigned to high- and low-PFKP groups, and differences in ESTIMATE-derived scores were compared using the 2-sided Mann–Whitney *U* test (*P* < 0.05). Violin plots were produced using the ggplot2 R package (version 3.3.6).

### CIBERSORT immune cell infiltration analysis

The relative proportions of 22 immune cell subtypes in HCC were estimated using the Cell-type Identification By Estimating Relative Subsets Of RNA Transcripts (CIBERSORT) algorithm with the LM22 signature matrix and 1,000 permutations. The analysis was performed using a local R script provided by the CIBERSORT platform (https://cibersort.stanford.edu), with quantile normalization set to Boolean false value. Only samples with CIBERSORT *P* values < 0.05 were retained for subsequent analyses. Correlations between immune cell proportions and PFKP expression were evaluated using either Spearman or Pearson correlation coefficients. Lollipop correlation plots were generated using a combination of ggplot2 and ggpubr.

### Immune checkpoint gene correlation analysis

A total of 47 immune checkpoint-related genes were selected based on published literature and the ImmPort database (accessed in December 2023). Pearson correlation analysis was performed in HCC samples to assess the associations between PFKP expression and the expression of these genes using the psych R package (version 2.2.9). *P* values were adjusted for multiple testing using the Benjamini–Hochberg method. Genes showing significant correlations were visualized in a heatmap generated with the pheatmap package (version 1.0.12).

### Immunophenoscore and immunotherapy response prediction

Immunotherapy response prediction data were obtained from The Cancer Immunome Atlas in December 2023. The immunophenoscore (IPS) for HCC samples was extracted and matched with gene expression data. Differences in IPS total scores and PD-1/CTLA-4-related scores between high-PFKP and low-PFKP expression groups were assessed using the Mann–Whitney *U* test. The results were visualized using box plots.

### Drug sensitivity prediction analysis

Drug sensitivity (IC_50_) of HCC samples was predicted using the pRRophetic R package (version 0.5) based on compounds from the Genomics of Drug Sensitivity in Cancer (GDSC) database. Log_2_(TPM + 1)-transformed expression data were used as input, with batch effects corrected using the ComBat method. The predictive model was trained on the GDSC1 dataset. Differences in predicted IC_50_ values between high-PFKP and low-PFKP expression groups were evaluated using 2-tailed Mann–Whitney *U* tests with FDR correction (significance threshold: FDR < 0.05).

### Tumor mutation burden analysis

Somatic mutation profiles for the TCGA-LIHC cohort were retrieved from the GDC Data Portal in December 2023 (https://portal.gdc.cancer.gov/, MC3 merged dataset, *.maf.gz format, GRCh38 reference genome). Mutation annotation followed the GDC standard pipeline, and only variants with a “PASS” filter status were retained. Tumor mutation burden (TMB) was defined as the number of nonsynonymous mutations per megabase, including missense, nonsense, frameshift insertions/deletions, splice-site, and nonstop mutations. TMB was calculated using the tmb() function in the maftools R package (version 2.14.0), assuming a total exonic length of 38 Mb, and was reported as mutations per megabase for each sample. For samples with matched expression and mutation data, the relationship between TMB and PFKP expression was evaluated using Spearman correlation analysis. PFKP expression data were transformed using log_2_(TPM+1), while TMB values were left untransformed. Correlation analysis was performed using the cor.test() function, and *P* < 0.05 was considered statistically significant.

Data visualization was conducted using ggplot2 (version 3.3.6), including scatter plots with fitted regression lines and box plots. In addition, samples were stratified into high-PFKP and low-PFKP expression groups according to the median PFKP level, and differences in TMB between these groups were compared using the Mann–Whitney *U* test.

### Clinical correlation analysis and prognostic modeling

Clinical data for the TCGA-LIHC cohort (n = 374) were downloaded from the TCGA GDC Data Portal (https://portal.gdc.cancer.gov/, accessed December 2023), including age, sex, tumor grade, American Joint Committee on Cancer (AJCC) stage, tumor-node-metastasis (TNM) classification, overall survival (OS), and follow-up time. For variables with partial missing values (e.g., TNM stage), multiple imputation was performed using the mice R package (version 3.14.0) with predictive mean matching (m = 5). All other analyses were conducted using complete-case data.

Patients were categorized into high-PFKP and low-PFKP expression groups according to the median expression value of PFKP. Differences in PFKP expression across clinical subgroups, including age, sex, tumor grade, and AJCC stage, were illustrated with box plots generated in ggplot2 (version 3.3.6). For variables that did not follow a normal distribution, between-group comparisons were performed using the Mann–Whitney *U* test. Associations between PFKP expression and clinicopathological characteristics were further displayed in heatmaps generated with pheatmap (version 1.0.12) based on z score-standardized input data. Multiple-testing correction was performed with the Benjamini–Hochberg method to control the FDR.

Survival endpoints included OS and progression-free survival, both measured in months. Patients were grouped according to the median PFKP expression level, and an optimal cut-off value was determined when applicable using the surv_cutpoint() function in the survminer package. Kaplan-Meier curves were fitted with survfit() and visualized using ggsurvplot(), and group differences were tested with the log-rank method.

Time-dependent receiver operating characteristic (ROC) curves were generated using the timeROC package (version 0.4) to evaluate the prognostic performance of PFKP expression for 1-, 3-, and 5-year OS. Area under the curve values and 95% confidence intervals (CIs) were calculated. Predictive performance was assessed internally without external validation.

Univariate and multivariate Cox proportional hazards regression analyses were performed using the survival R package (version 3.5-5). Variables included PFKP expression (high vs low), age (continuous), sex (male vs female), tumor grade (G1-G4, dummy-coded), and AJCC stage (I-IV, dummy-coded). Multicollinearity was assessed using variance inflation factors, and the proportional hazards assumption was evaluated using cox.zph(), with *P* > 0.05 indicating no significant violation.

A nomogram for 1-, 3-, and 5-year survival prediction was constructed based on the multivariate Cox model using the rms package (version 6.5-0). Internal validation was performed using bootstrap resampling (n = 1,000). Calibration curves were generated using calibrate(), and the concordance index, along with its 95% CI, was reported to assess model performance.

### Immunohistochemistry

Clinical tissue specimens were fixed in 4% neutral formalin for 24 hours, paraffin-embedded, and cut into 4 μm sections. After dewaxing in xylene and rehydration through a graded ethanol series, antigen retrieval was performed in citrate buffer (pH 6.0) by microwave heating at 95 °C for 10 minutes. Endogenous peroxidase activity was blocked with 3% hydrogen peroxide at room temperature for 20 minutes, followed by blocking with 5% goat serum (ZSGB-BIO, Cat#SP-9001) for 10 minutes.

Primary antibodies against PFKP (Boster, clone OTI1F2, 1:100, diluted in 1% bovine serum albumin [BSA]) and Ki67 (Maixin, Cat#RMA-0542, 1:500, diluted in 1% BSA) were applied overnight at 4 °C for 12–16 hours. After PBS washing, sections were incubated with a biotin-conjugated secondary antibody (ZSGB-BIO, Cat#SAP-9100) for 30 minutes at room temperature. Signal detection was performed using a 3,3'-diaminobenzidine kit (ZSGB-BIO, Cat#ZLI-9017), followed by hematoxylin counterstaining for 5 minutes. The sections were subsequently dehydrated, cleared, sealed, and examined microscopically. Images were acquired at a standardized magnification of 400×. All slides were independently reviewed in a blinded manner by 2 senior pathologists.

Immunostaining was semiquantitatively scored using the *H*-score formula: *H*-score = Σ (staining intensity × percentage of positive cells), where staining intensity ranged from 0 to 3 and the proportion of positive cells ranged from 0% to 100%. Each experimental group included at least 3 independent clinical samples.

### Cell culture and treatment

The human HCC cell line HepG2 (purchased from the Cell Bank of the Chinese Academy of Sciences) was cultured in high-glucose Dulbecco's Modified Eagle Medium (Gibco, Cat#C11995500CP) supplemented with 10% fetal bovine serum (Gibco, Cat#10270106), 100 U/mL penicillin, and 100 μg/mL streptomycin (Gibco, Cat#15140122). Cells were maintained in a humidified incubator at 37 °C with 5% CO_2_. Before experimentation, cells were authenticated by short tandem repeat profiling and confirmed to be free of mycoplasma contamination. Only cells with passage numbers between 5 and 10 were used.

### PFKP overexpression and rescue experiment

To generate a stable PFKP-overexpressing model, HepG2 cells were transfected with a recombinant plasmid carrying full-length human PFKP cDNA (pcDNA3.1-PFKP) using Lipofectamine 3000 (Invitrogen, Cat#L3000015). Transfected cells were selected with G418 (600 μg/mL; Sigma, Cat#G8168) for 14 days, and stable clones were expanded for further analysis. To examine the glycolysis-dependent mechanism, rescue experiments were performed by treating PFKP-overexpressing cells with 2-deoxy-d-glucose (2-DG; Sigma, Cat#D8375) at a final concentration of 10 mM for 24 hours. The experimental groups were defined as follows: PFKP-negative control, empty vector control; PFKP-OE, PFKP overexpression; and PFKP-OE+2-DG, PFKP overexpression with 2-DG treatment (10 mM, 24 hours).

### Western Blot

Cells from each experimental group were washed 3 times with prechilled PBS and lysed using radioimmunoprecipitation assay buffer (Beyotime, Cat#P0013B), protease inhibitors (Roche, Cat#11873580001), and phosphatase inhibitors (Thermo, Cat#78420). After incubation on ice for 30 minutes with intermittent vortexing, lysates were centrifuged at 12,000*g* for 15 minutes, and the supernatants were collected. Protein concentrations were measured using a bicinchoninic acid protein assay kit (Beyotime, Cat#P0012), and all samples were adjusted to 3 μg/μL. Equal amounts of protein (30 μg per lane) were separated by sodium dodecyl sulfate-polyacrylamide gel electrophoresis and transferred onto polyvinylidene difluoride membranes (Millipore, Cat#IPVH00010) at 100 V. Membranes were blocked with 5% nonfat milk in Tris-buffered saline with Tween 20 for 1 hour and incubated overnight at 4 °C with the indicated primary antibodies (listed in Table S1, http://links.lww.com/CTG/B521). The following day, membranes were incubated for 1 hour at room temperature with HRP-conjugated goat anti-rabbit IgG (Proteintech, Cat#SA00001-2, 1:5,000) or goat anti-mouse IgG (Proteintech, Cat#SA00001-1, 1:5,000), depending on the host species of the primary antibody. Protein bands were developed with an enhanced chemiluminescence reagent (Beyotime, Cat#P0018FS) and captured using a Tanon 5,200 imaging system. Band density was analyzed in ImageJ, with glyceraldehyde-3-phosphate dehydrogenase serving as the internal loading reference. At least 3 independent biological replicates were included in each group.

### Statistical analysis

**Figure 1.**
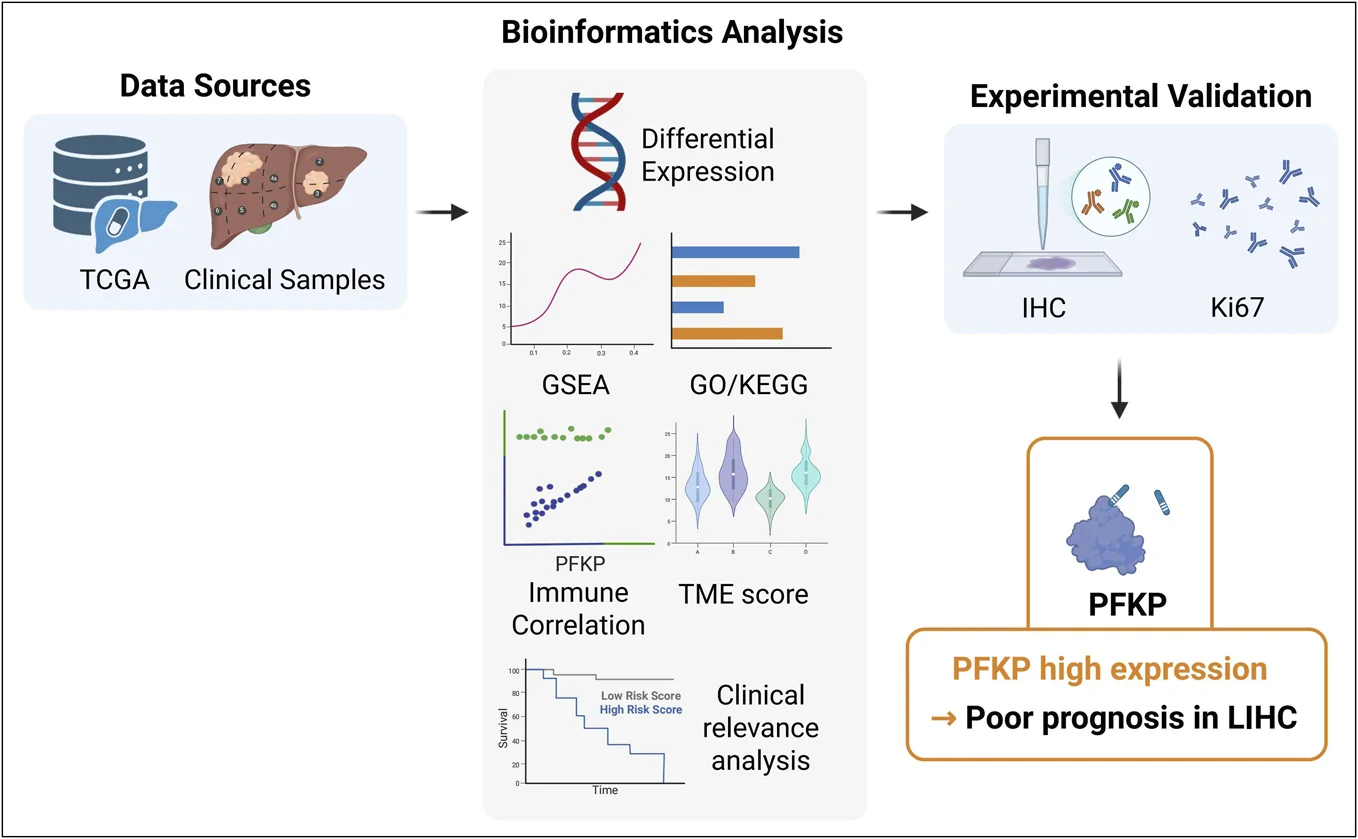
Overall flowchart of the analytical process. GO, gene ontology; GSEA, gene set enrichment analysis; KEGG, Kyoto Encyclopedia of Genes and Genomes; IHC, immunohistochemistry; PFKP, platelet-type phosphofructokinase; TCGA, The Cancer Genome Atlas; TME, tumor microenvironment.

Clinical data and gene expression matrices were preprocessed using custom Perl scripts (Strawberry Perl, version 5.30.0) for clinical information extraction, format conversion, and sample matching. All statistical analyses and data visualizations were performed in R (version 4.2.1; R Foundation for Statistical Computing, Vienna, Austria) on a Windows 10 platform. Immunohistochemical images were independently evaluated by 2 pathologists. Positive staining was quantified using ImageJ on the basis of either the *H*-score method or automated segmentation with the Color Deconvolution plugin, and values from multiple regions were averaged for analysis. All statistical analyses were conducted using 2-sided tests, with *P* < 0.05 considered statistically significant. For multiple-testing correction, the Benjamini–Hochberg procedure was applied to control the FDR. Data with a normal distribution are presented as mean ± SD, whereas non-normally distributed data are summarized as median and interquartile range. The major R packages used and their corresponding analytical tasks are listed in Table 1, and the complete analytical workflow is illustrated in Figure 1.

**Table 1.** Summary of R packages used in various stages of this study

Process	Packages
Analysis of gene expression and function	ggplot2, stats, car, limma, ggpubr, ggExtra, circlize, corrplot, clusterProfiler, org.Hs.eg.db, enrichplot, circlize, RColorBrewer, dplyr, ComplexHeatmap, enrichplot
Gene set enrichment analysis	limma, org.Hs.eg.db, clusterProfiler, enrichplot
Study on the correlation between TME and immunity	limma, estimate, reshape2, ggpubr, CIBERSORT, ggpubr, vioplot, ggExtra, ggplot2, corrplot, pRRophetic
Tumor mutation burden	limma, ggplot2, ggpubr, ggExtra
Clinical correlation analysis and prognosis assessment	limma, ggpubr, ComplexHeatmap, survival, survminer, timeROC, regplot, rms

## RESULTS

### PFKP is significantly overexpressed in HCC tissues

**Figure 2.**
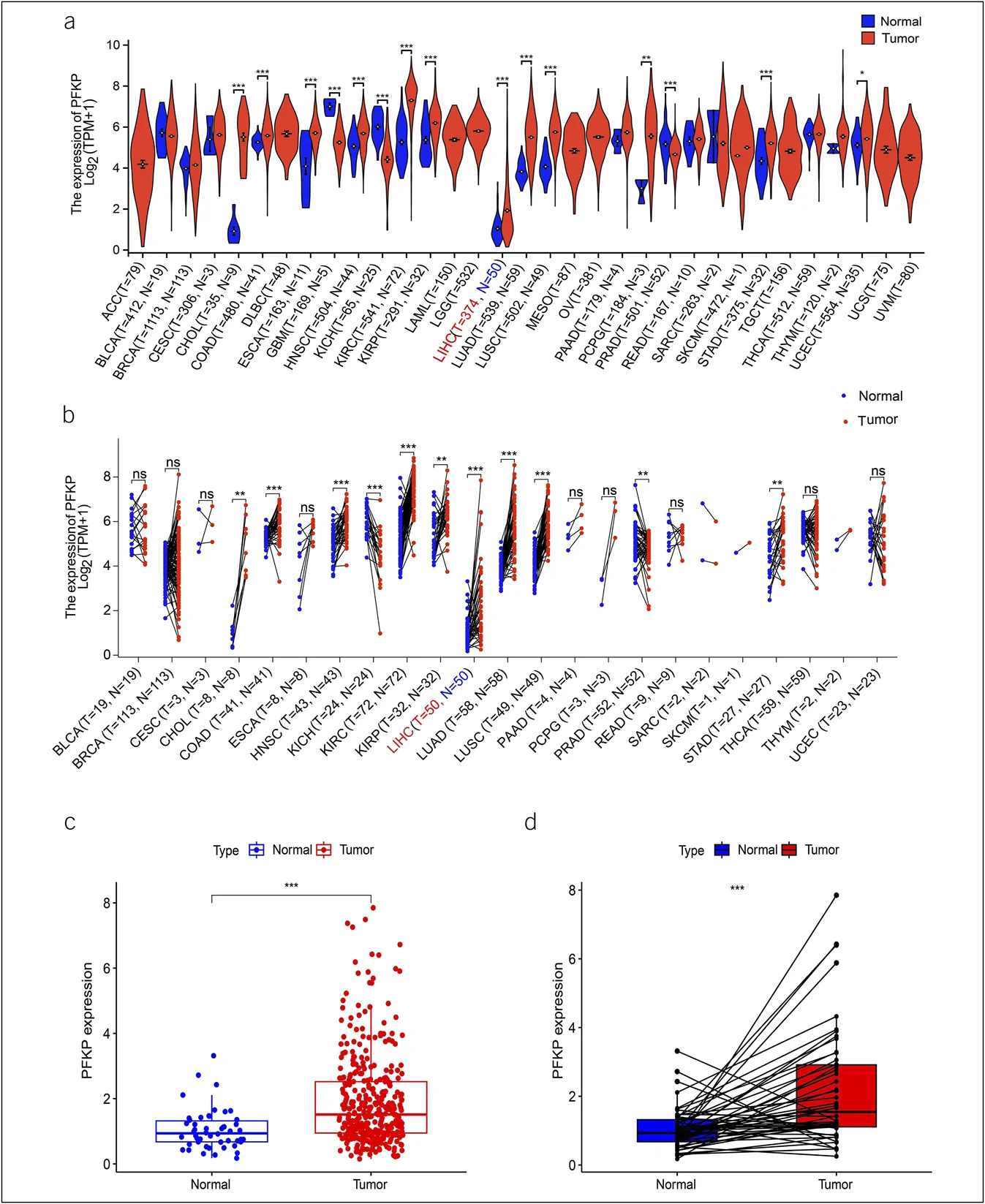
Expression analysis of PFKP in HCC and adjacent normal tissues based on the TCGA database. (**a**) PFKP expression across pan-cancer; (**b**) PFKP expression in paired tumor and normal tissues across multiple cancer types; (**c**) differential expression of PFKP between HCC tumor tissues and normal tissues; (**d**) differential expression of PFKP in paired HCC tumor and adjacent normal samples. Expression levels are presented as log_2_-transformed TPM values. Statistical methods: The Mann–Whitney *U* test was used for unpaired samples and the Wilcoxon signed-rank test for paired samples. HCC, hepatocellular carcinoma; PFKP, platelet-type phosphofructokinase; TCGA, The Cancer Genome Atlas. Significance annotations: NS, *P* ≥ 0.05; *P < 0.05; **P < 0.01; ***P < 0.001.

Pan-cancer analysis of TCGA data showed that PFKP was upregulated in most tumor types, except for glioblastoma multiforme, kidney chromophobe, and prostate adenocarcinoma, and was notably elevated in HCC (Figure 2a). Within the TCGA-LIHC cohort, PFKP levels were markedly higher in 374 HCC samples than in 50 normal liver tissues (*P* < 0.001, Figure 2c). Consistently, paired-sample analysis also showed significantly increased PFKP expression in HCC tissues relative to matched adjacent normal tissues (*P* < 0.001, Figure 2b,d). These results indicate that PFKP is consistently overexpressed in HCC tissues.

### High PFKP expression is significantly associated with poor prognosis and advanced T stage

**Figure 3.**
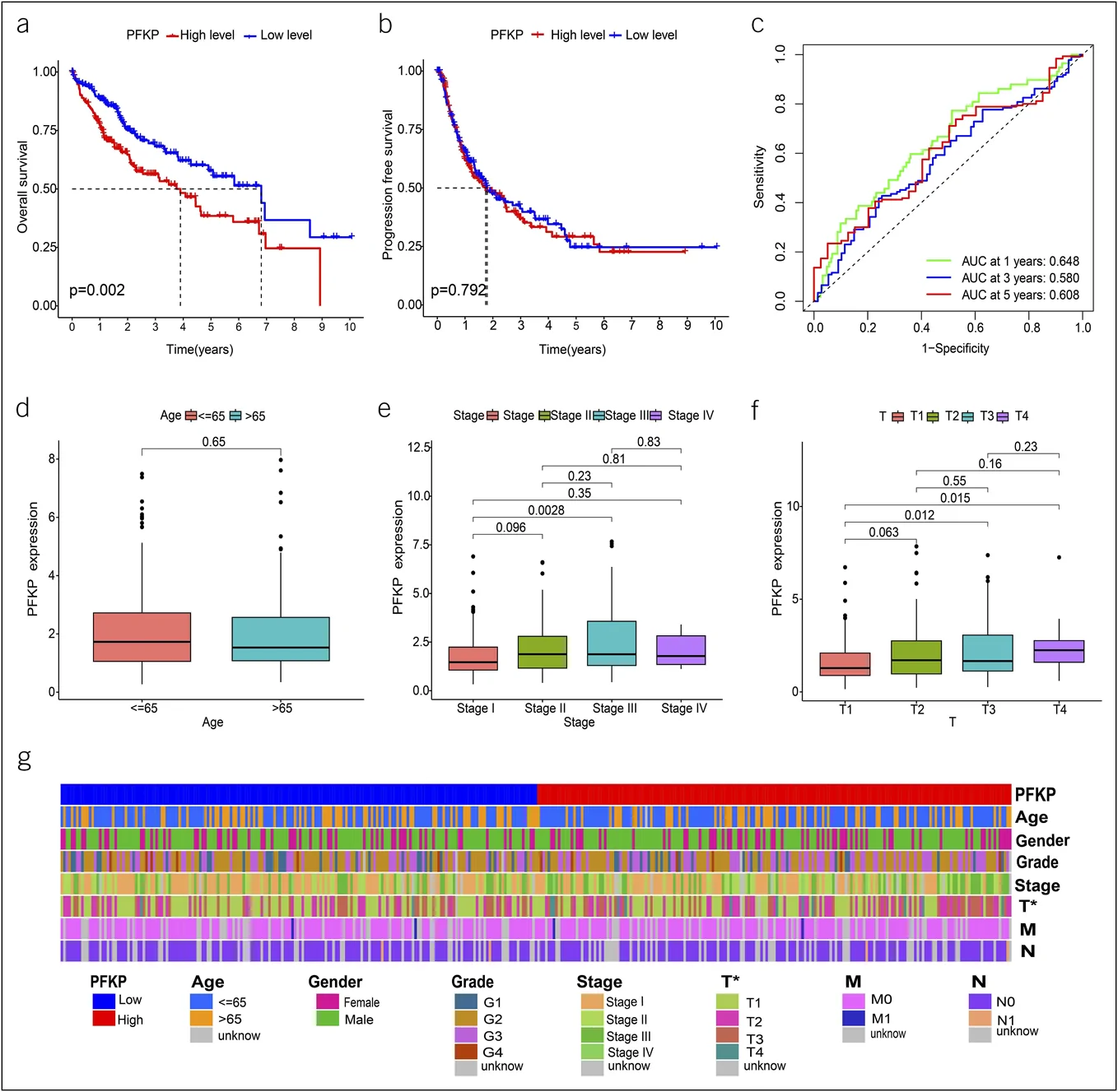
Correlation analysis between PFKP expression, prognosis, and clinicopathological features in hepatocellular carcinoma. (**a**) Kaplan-Meier OS curve; (**b**) Kaplan-Meier progression-free survival curve; (**c**) receiver operating characteristic curves and corresponding AUC values for 1-, 3-, and 5-year OS prediction; (**d**) comparison of PFKP expression among different age groups; (**e**) PFKP expression across different AJCC stages; (**f**) PFKP expression across different *T* stages; (**g**) integrated heatmap of PFKP expression levels and clinicopathological features. Statistical analysis: The log-rank test was applied for survival analysis; the Wilcoxon rank-sum test was used for group comparisons. AJCC, American Joint Committee on Cancer; AUC, area under the curve; OS, overall survival; PFKP, platelet-type phosphofructokinase.

Kaplan-Meier analysis indicated that patients with elevated PFKP expression experienced significantly shorter OS than those with lower expression (hazard ratio [HR] = 1.224, 95% CI: 1.089–1.374, *P* = 0.002, Figure 3a), while no significant difference was detected in progression-free survival (*P* = 0.792, Figure 3b). ROC curve analysis indicated limited predictive value of PFKP for 1-, 3-, and 5-year survival, with area under the curves of 0.648, 0.580, and 0.608, respectively (Figure 3c).

Clinical subgroup analysis showed no significant difference in PFKP expression between patients aged 65 or older and younger than 65 years (*P* = 0.65, Figure 3d). Among AJCC stages, a significant difference was observed only between stage I and stage III tumors (*P* = 0.0028, Figure 3e). In the TNM classification, PFKP expression was significantly higher in T3 and T4 tumors than in T1 tumors (T1 vs T3, *P* = 0.012; T1 vs T4, *P* = 0.015; Figure 3f).

Integrated heatmap analysis further showed that among the examined clinical variables, only T stage differed significantly between the high-PFKP and low-PFKP groups (Figure 3g), indicating a closer relationship between PFKP expression and tumor invasive status. Taken together, these findings suggest that elevated PFKP expression is associated with unfavorable OS and more advanced T stage in HCC.

### PFKP is an independent prognostic factor for OS in HCC

**Figure 4.**
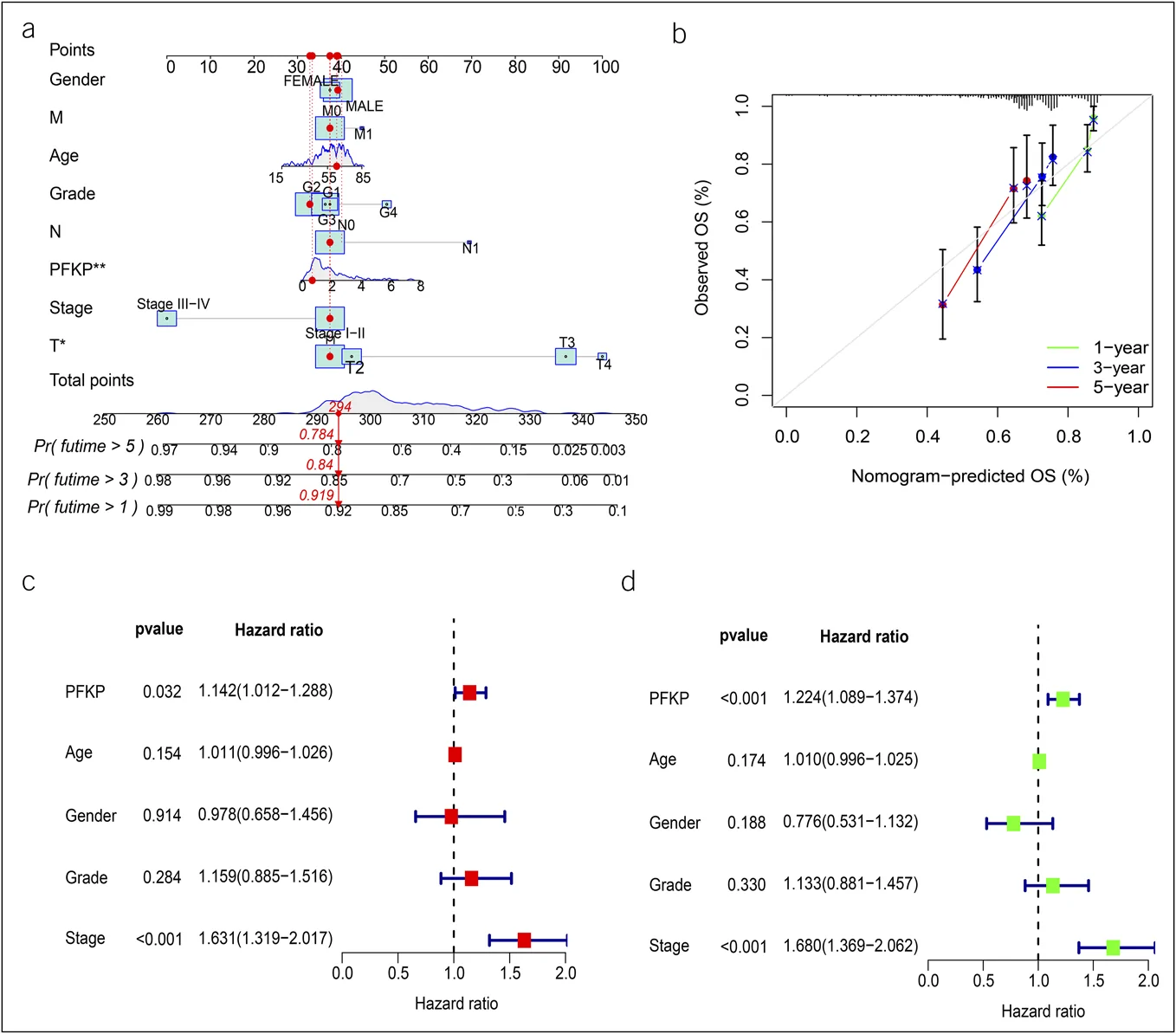
PFKP serves as an independent prognostic factor in HCC. (**a**) Representative patient: ID TCGA-DD-AADS; age: 63 years; sex: male; stage: Stage I; T1M0N0; PFKP expression level: 0.710. The total nomogram score was 294. The predicted 1-, 3-, and 5-year OS probabilities were 0.919, 0.84, and 0.784, respectively; the actual survival time was 1.30 years; (**b**) Calibration plot. The x-axis represents the predicted probability of OS, and the y-axis shows the observed OS probability in HCC patients. The grey dashed line indicates the ideal reference line; (**c**) Univariate Cox regression analysis of prognostic factors; (**d**) Multivariate Cox regression analysis of prognostic factors. HCC, hepatocellular carcinoma; OS, overall survival; PFKP, platelet-type phosphofructokinase.

To assess the prognostic relevance of PFKP for OS in HCC, a Cox regression model incorporating PFKP expression and clinical variables was established. Based on this model, a nomogram was constructed to estimate the probabilities of 1-, 3-, and 5-year OS (Figure 4a). For example, a 63-year-old male patient (TCGA-DD-AADS; stage I; T1M0N0; PFKP expression = 0.710) had a total risk score of 294, corresponding to predicted 1-, 3-, and 5-year survival probabilities of 0.919, 0.840, and 0.784, respectively. The observed survival time for this patient was 1.30 years. Calibration analysis showed good agreement between the predicted and observed survival outcomes (Figure 4b).

In univariate Cox analysis, PFKP expression (HR = 1.142, 95% CI: 1.012–1.288, *P* = 0.032) and AJCC stage (HR = 1.631, 95% CI: 1.319–2.017, *P* < 0.001) were both significantly related to OS (Figure 4c). Multivariate analysis further showed that PFKP expression remained independently associated with OS (HR = 1.224, 95% CI: 1.089–1.374, *P* < 0.001), together with AJCC stage (Figure 4d).

Taken together, these findings suggest that PFKP has independent prognostic relevance in HCC and may serve as a candidate biomarker for survival stratification.

### GO/KEGG functional enrichment and GSEA analyses of PFKP coexpressed genes

**Figure 5.**
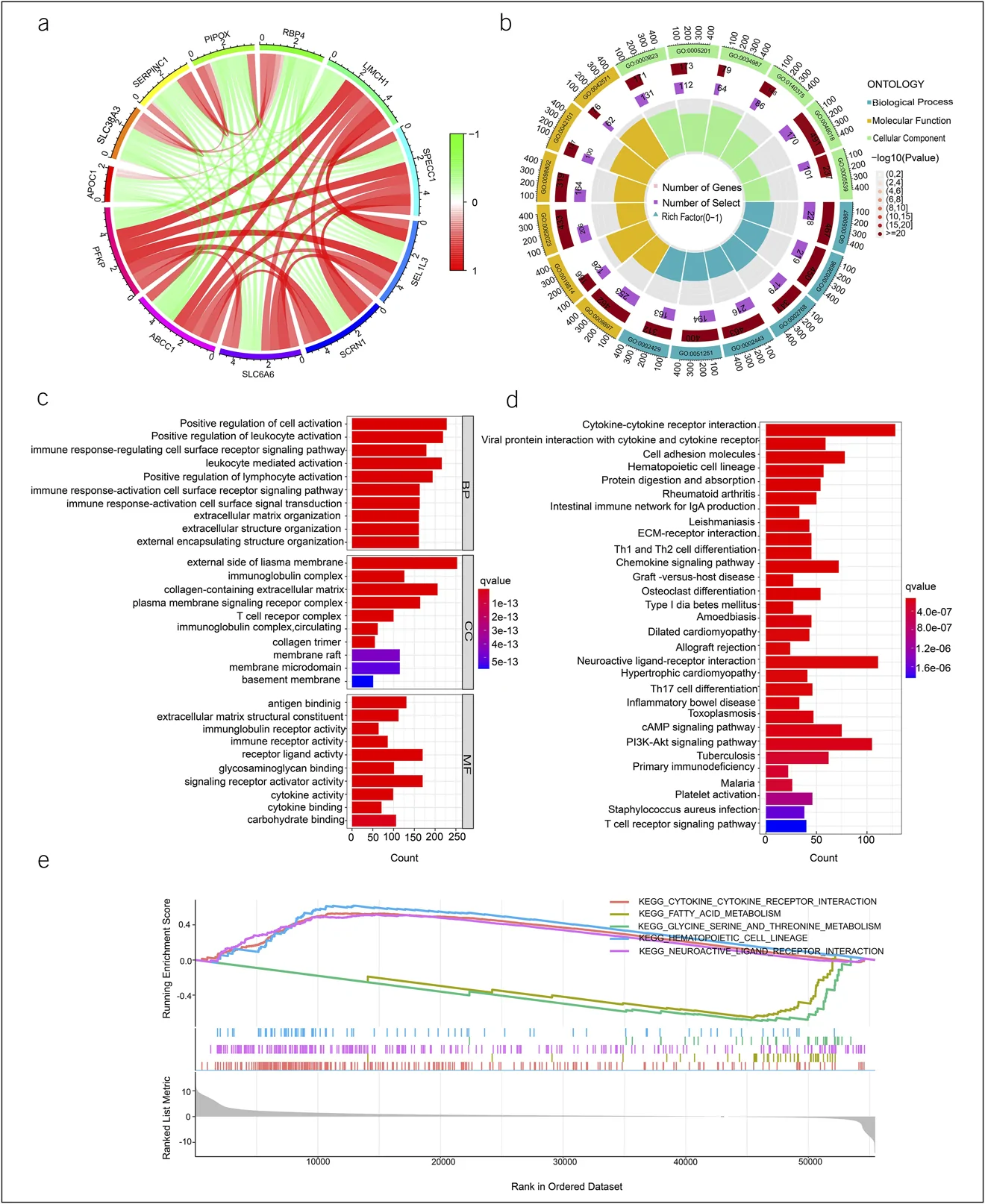
Functional enrichment analysis of PFKP in hepatocellular carcinoma. (**a**) Chord plot showing the top 10 genes coexpressed with PFKP. The outer ring displays gene names; red lines indicate positive correlations with PFKP; and green lines indicate negative correlations; (**b**) Circular visualization of GO enrichment results; (**c**) bar plot of GO enrichment terms; (**d**) bar plot of KEGG enrichment pathways; (**e**) GSEA enrichment analysis comparing high vs low PFKP expression groups. Statistical criteria: Differential gene selection was based on |logFC| > 1 and FDR < 0.05. Enrichment analysis was performed using the clusterProfiler package (version 4.2.2) with gene sets from MSigDB KEGG (version 7.5.1). GSEA was conducted with 1,000 permutations, and statistical significance was defined as FDR < 0.25. FDR, false discovery rate; GO, gene ontology; GSEA, gene set enrichment analysis; KEGG, Kyoto Encyclopedia of Genes and Genomes; PFKP, platelet-type phosphofructokinase.

To explore the potential biological roles of PFKP in HCC, we identified the top 10 genes most strongly correlated with PFKP expression using Pearson correlation analysis (|cor| > 0.6, *P <* 0.001) and visualized their relationships in a chord diagram (Figure 5a). PFKP expression was positively correlated with genes including LIMCH1, SPECC1, SEL1L3, and SCRN1, whereas negative correlations were observed with genes such as APOC1 and SLC38A3.

Differential expression analysis yielded a large number of differentially expressed genes (criteria: |logFC| > 1, FDR < 0.05). GO enrichment analysis revealed that these genes were primarily involved in immune-related BP, such as lymphocyte activation, immunoglobulin complex formation, and antigen binding (Figure 5b,c). KEGG pathway analysis identified significant enrichment in cytokine-cytokine receptor interaction, PI3K-Akt signaling, and neuroactive ligand-receptor interaction pathways (Figure 5d).

GSEA demonstrated that the high-PFKP group was enriched for immune-related pathways, including hematopoietic cell lineage and cytokine-cytokine receptor interaction, whereas the low-PFKP group showed enrichment of pathways associated with glycine, serine, and threonine metabolism as well as fatty acid metabolism (Figure 5e). These results suggest that PFKP may be linked to both immune regulation and metabolic remodeling in HCC.

### Correlation between PFKP expression, TME, and immune checkpoint genes

**Figure 6.**
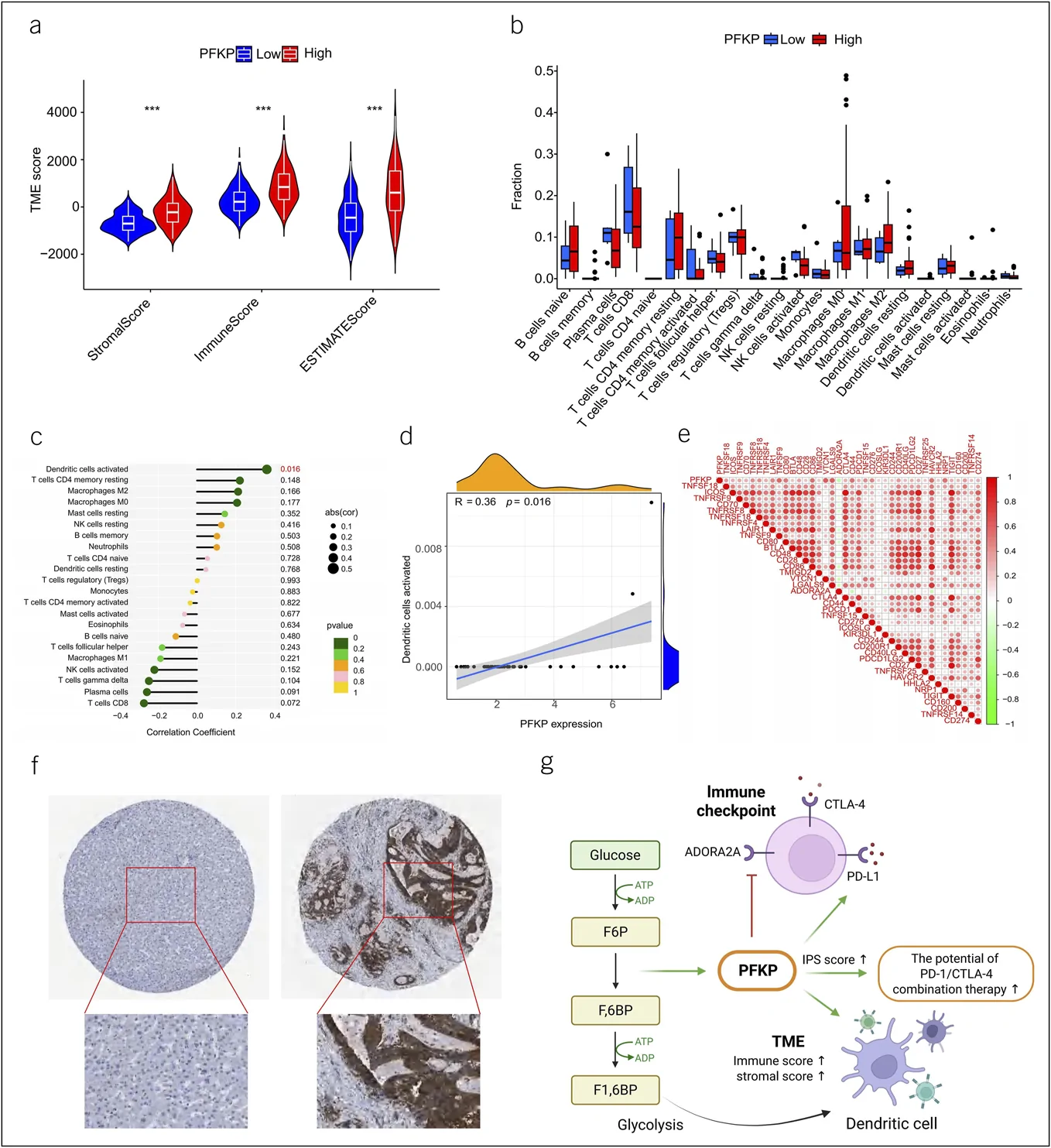
Association between PFKP expression and the TME. (**a**) Correlation between PFKP expression and TME scores (Wilcoxon test); (**b**) distribution of immune cell infiltration between high and low PFKP expression groups; (**c**) correlation analysis between PFKP expression and immune cell populations (Spearman correlation); (**d**) positive correlation between PFKP expression and activated dendritic cells; (**e**) heatmap showing correlations between PFKP and immune checkpoint-related genes (n = 374, Spearman correlation); (**f**) IHC images from the human protein atlas database: left panel, normal liver tissue; right panel, hepatocellular carcinoma tissue; (**g**) Schematic illustration of PFKP-mediated immune regulation mechanisms. PFKP, platelet-type phosphofructokinase.

To evaluate the association between PFKP expression and the TME, we applied the ESTIMATE algorithm to characterize the immune and stromal components of HCC samples. The high-PFKP expression group showed significantly higher immune, stromal, and ESTIMATE scores than the low-expression group (all *P <* 0.001; Figure 6a), indicating a more complex microenvironmental composition.

Among the 22 immune cell subsets estimated by CIBERSORT, tumors with high PFKP expression exhibited increased infiltration of activated dendritic cells, memory CD4^+^ T cells, and M2 macrophages (Figure 6b,c). Correlation analysis further showed that PFKP expression was most strongly associated with activated dendritic cells (R = 0.36, *P* = 0.016; Figure 6d).

Analysis of immune checkpoint genes showed that PFKP expression was positively correlated with several canonical checkpoint molecules, including PDCD1 (PD-1), CTLA4, LAG3, and T cell immunoreceptor with Ig and ITIM domains, whereas a negative correlation was observed with ADORA2A (Figure 6e). IHC staining confirmed elevated PFKP expression in tumor tissues (Figure 6f).

Collectively, as illustrated in Figure 6g, high PFKP expression was associated with a more immune-infiltrated yet checkpoint-enriched microenvironment, suggestive of an immunoregulatory or potentially immunosuppressive state. These findings suggest that PFKP may be involved in shaping the immune contexture of HCC and may have relevance to immunotherapeutic responsiveness.

### High PFKP expression is associated with predicted immunotherapy responsiveness and drug sensitivity

**Figure 7.**
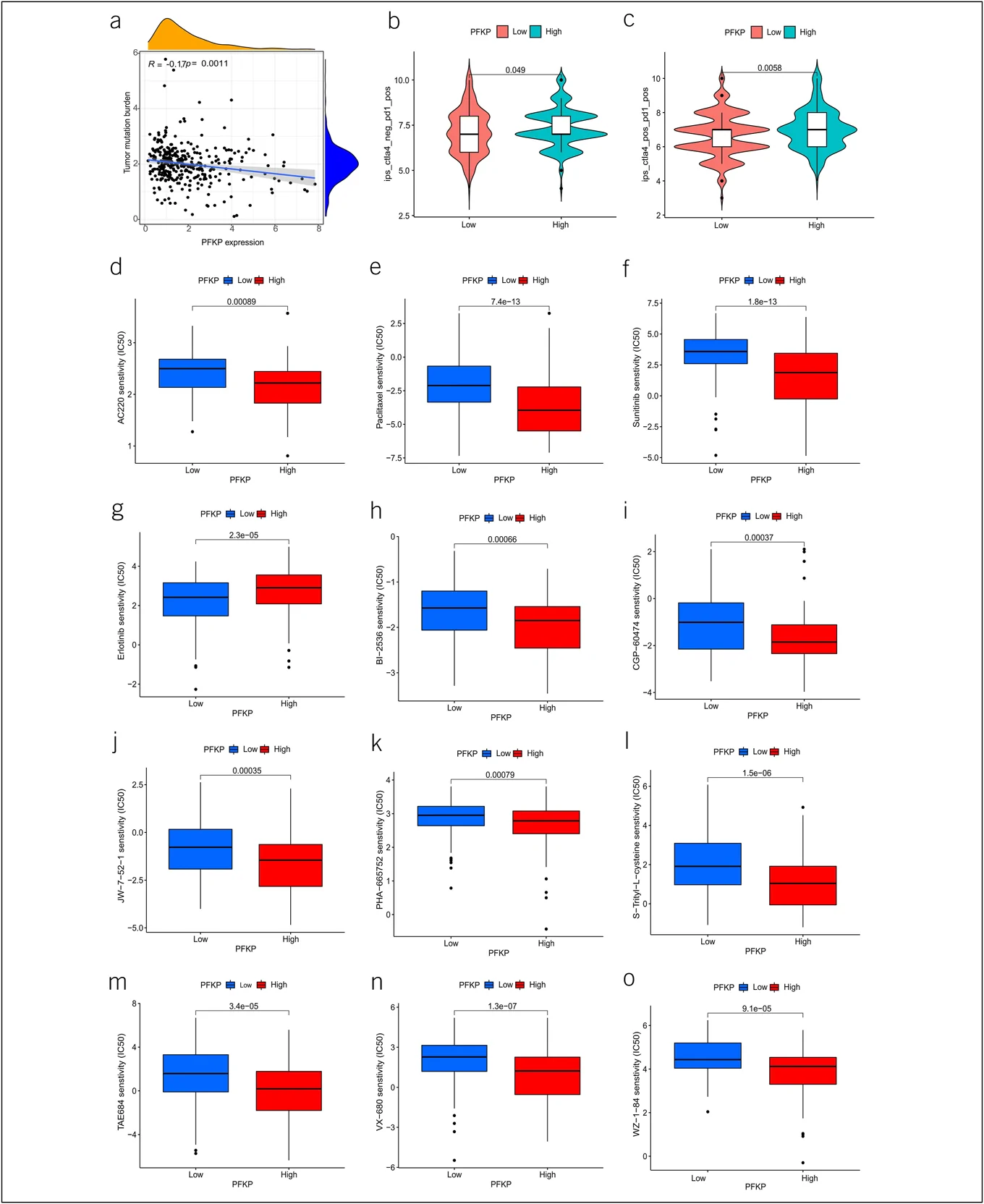
Association of PFKP expression with immunotherapy response and drug sensitivity in hepatocellular carcinoma. (**a**) Correlation between PFKP expression and TMB (Spearman correlation); (**b** and **c**) association of PFKP expression with predicted immunotherapy response, as assessed by IPS scores (Wilcoxon test); (**d**–**o**) comparison of predicted drug sensitivity/IC50 between high- and low-PFKP expression groups (IC_50_ prediction using the pRRophetic algorithm, Genomics of Drug Sensitivity in Cancer database): (**d**) quizartinib (AC220); (**e**) paclitaxel; (**f**) sunitinib; (**g**) erlotinib; (**h**) BI-2536; (**i**) CGP-60474; (**j**) JW-7-52-1; (**k**) PHA-665752; (**l**) S-Trityl-L-cysteine; (**m**) TAE684; (**n**) VX-680; (**o**) WZ-1-84. PFKP, platelet-type phosphofructokinase.

To explore the relationship between PFKP expression and immunotherapy-related features, we examined its association with TMB. Spearman correlation analysis showed a significant inverse correlation between PFKP expression and TMB (R = −0.17, *P* = 0.0011; Figure 7a), suggesting that its immunogenic potential may arise from nonmutational mechanisms.

We next compared immunotherapy-related scores between the high-PFKP and low-PFKP expression groups using IPS data from the The Cancer Immunome Atlas database. The high-PFKP expression group showed significantly higher IPS under both PD-1 blockade (*P* = 0.049) and combined PD-1 plus CTLA-4 blockade (*P* = 0.0058; Figure 7b,c), indicating a potentially greater sensitivity to ICIs.

To further assess the relationship between PFKP expression and drug response, we applied the pRRophetic algorithm to estimate IC_50_ values for drugs in the GDSC database. The high-PFKP group showed lower predicted IC_50_ values for quizartinib, paclitaxel, and sunitinib (Figure 7d–f), whereas the low-PFKP group showed lower predicted I_C50_ values for erlotinib (Figure 7g).

In addition, several compounds, including BI-2536, CGP-60474, and TAE684, also showed significantly reduced predicted IC_50_ values in the high-PFKP group (*P* < 0.001; Figure 7h–o). Taken together, these findings suggest that high PFKP expression is associated with predicted immunotherapy responsiveness and distinct drug sensitivity profiles, indicating that PFKP may serve as a candidate biomarker for therapeutic stratification in HCC.

### IHC validation of elevated PFKP expression and its association with immune checkpoint proteins in HCC

**Figure 8.**
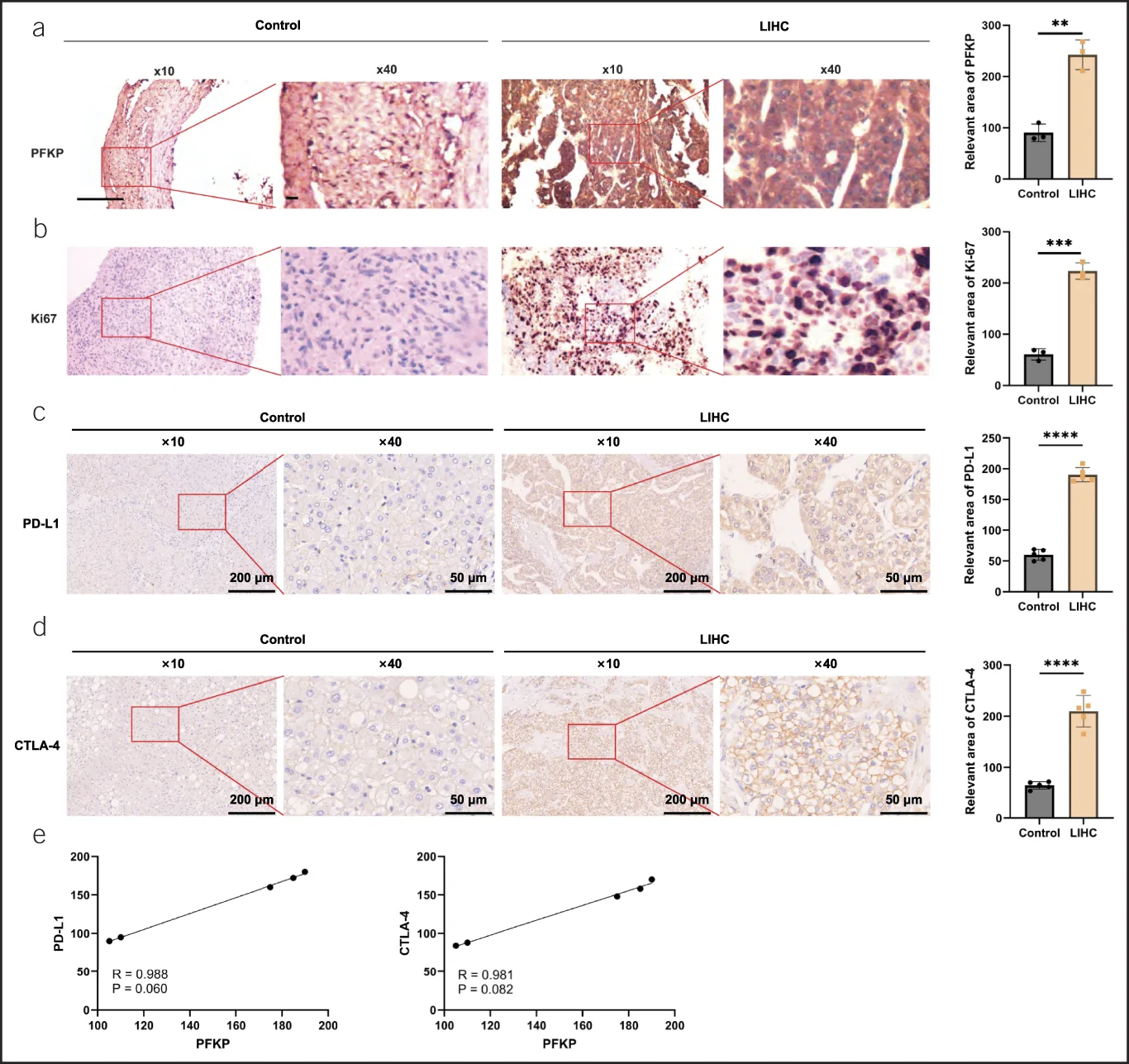
PFKP expression in HCC tissues and its association with cell proliferation and immune checkpoint proteins. (**a**) Representative IHC staining and quantitative analysis showing that PFKP expression was significantly elevated in HCC tissues compared with paired adjacent normal tissues.. Scale bars: 200 μm (×10); 50 μm (×40); (**b**) Representative IHC staining and quantitative analysis showing that Ki-67 expression was significantly elevated in HCC tissues compared with paired adjacent normal tissues (n = 5, **P < 0.01, ***P < 0.001); (**c** and **d**) in HCC tissues with high PFKP expression, PD-L1 and CTLA-4 protein levels were markedly upregulated. Scale bars: 200 μm (×10); 50 μm (×40); (**e**) PFKP expression was positively correlated with PD-L1 and CTLA-4 levels at the tissue level (R = 0.988, *P* = 0.060; R = 0.981, *P* = 0.082). HCC, hepatocellular carcinoma; PFKP, platelet-type phosphofructokinase.

To confirm PFKP expression at the protein level, we reviewed immunohistochemical evidence from the Human Protein Atlas, which indicated stronger PFKP staining in HCC tissues than in adjacent normal liver tissues (Figure 6f). We next performed IHC staining on clinical HCC specimens (n = 5) and matched adjacent normal tissues (n = 5) collected from the Second People's Hospital of Bengbu. In agreement with the Human Protein Atlas results, PFKP expression was significantly elevated in HCC tissues (*P* < 0.01; Figure 8a). In addition, the proliferation marker Ki-67 was also markedly elevated in HCC tissues (*P* < 0.001; Figure 8b), indicating enhanced proliferative activity in tumor samples. To further assess the relationship between PFKP and immune checkpoint molecules, parallel IHC staining was performed on the same clinical specimens. HCC tissues with high PFKP expression also showed higher PD-L1 and CTLA-4 protein levels (Figure 8c,d). Correlation analysis based on H-scores demonstrated that PFKP expression was positively correlated with PD-L1 (R = 0.988, *P* = 0.060) and CTLA-4 (R = 0.981, *P* = 0.082; Figure 8e). These findings suggest that elevated PFKP expression is associated with increased immune checkpoint expression in HCC and may be related to an immunoregulatory microenvironment.

### PFKP regulates the expression of immune-related factors through the glycolysis pathway

**Figure 9.**
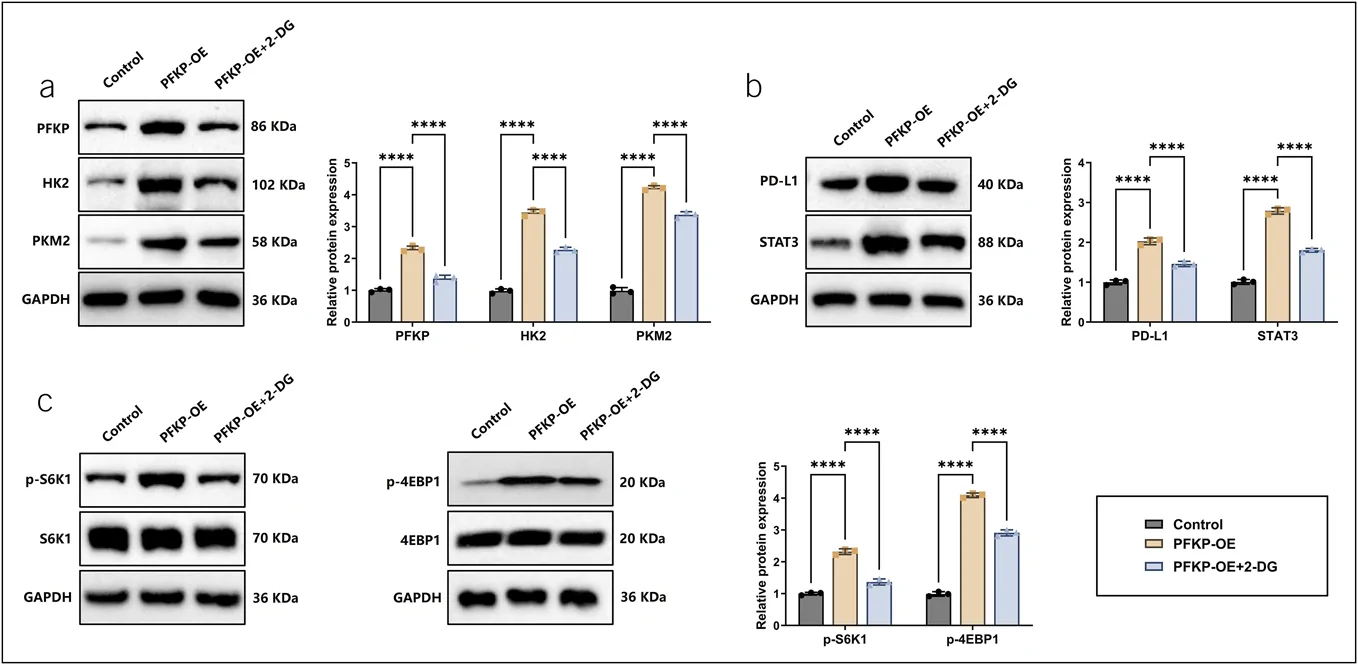
PFKP regulates immune-related protein expression and activates mTOR signaling through glycolysis. (**a**) Western Blot analysis of glycolysis-associated proteins upon PFKP overexpression; (**b**) expression profiles of immune-regulatory proteins; (**c**) analysis of downstream mTOR signaling pathway components. All protein expression levels were normalized and reported as “Relative protein expression” (n = 3, mean ± SD). Statistical analysis was conducted using one-way analysis of variance (ANOVA) followed by the Tukey multiple comparisons test. mTOR, mechanistic target of rapamycin; PFKP, platelet-type phosphofructokinase. *P < 0.05, **P < 0.01, ***P < 0.001.

To explore the role of PFKP in glycolysis and immune-related regulation, we established a PFKP-overexpressing HepG2 cell model and included 3 groups: control, PFKP overexpression (PFKP-OE), and PFKP overexpression combined with 2-DG treatment (10 mM; PFKP-OE+2-DG). Western Blot analysis showed that PFKP overexpression significantly increased PFKP protein levels and was accompanied by upregulation of the glycolytic enzymes HK2 and PKM2 (*P* < 0.05; Figure 9a). By contrast, 2-DG treatment markedly reduced the expression of these proteins, suggesting that the effects associated with PFKP overexpression were at least partly dependent on glycolytic activity.

Further analysis showed that PFKP overexpression significantly increased the expression of PD-L1 (*P* < 0.001) and STAT3 (*P* < 0.001; Figure 9b). These increases were attenuated in the PFKP-OE+2-DG group, with protein levels approaching those of the control group, suggesting that glycolytic activity may be involved in the upregulation of these immune-related factors.

To further assess pathway involvement, we examined 2 downstream phosphorylation markers of mTOR signaling, p-S6K1, and p-4EBP1. PFKP overexpression significantly increased the phosphorylation levels of both proteins (*P* < 0.001), whereas 2-DG treatment effectively suppressed this activation (Figure 9c). These results suggest that PFKP overexpression is associated with activation of mTOR-related signaling in a glycolysis-dependent manner.

In summary, these findings suggest that PFKP may participate in shaping the HCC microenvironment by enhancing glycolytic activity and modulating mTOR-related signaling, thereby contributing to the expression of immune checkpoint-related molecules.

## DISCUSSION

This study systematically characterized the expression pattern and clinical relevance of PFKP in HCC. By integrating TCGA-based analyses with immunohistochemical validation, we found that PFKP was significantly overexpressed in HCC tissues and was associated with poorer OS, supporting its potential prognostic relevance. Previous studies have shown that PFKP, as a key rate-limiting enzyme in glycolysis, participates in metabolic reprogramming and tumor progression in several malignancies (16,23). However, its role in HCC has not been fully defined. To our knowledge, this study investigates the association between PFKP expression and clinical outcomes in HCC and incorporates PFKP into a nomogram for individualized survival prediction. Compared with conventional clinicopathological indicators such as TNM staging, PFKP may provide additional biologically relevant information for risk stratification. These findings offer insight into the potential value of PFKP in metabolic characterization and personalized management of HCC.

In addition, our data suggest that PFKP overexpression is associated with enhanced glycolytic activity and activation of mTOR-related signaling. Specifically, the glycolytic enzymes HK2 and PKM2 were upregulated, accompanied by increased phosphorylation of p-S6K1 and p-4EBP1. These changes were attenuated after 2-DG treatment, indicating that the observed effects were at least partly dependent on glycolytic activity. Previous studies on glycolysis-mTOR crosstalk have mainly focused on upstream regulators such as AKT or HIF-1α (24,25). By contrast, our findings suggest that PFKP may also be involved in linking glycolytic reprogramming to mTOR-related signaling. This extends the potential functional relevance of PFKP beyond metabolic enzyme activity alone and supports the view that PFKP may participate in coordinating metabolic and signaling events in HCC.

In the context of the TME, elevated PFKP expression was accompanied by increased TME scores and showed a positive correlation with the infiltration of immune cells such as activated dendritic cells. Several immune checkpoint molecules, including PD-L1, CTLA-4, and CD274, were significantly upregulated in tumors with high PFKP expression, suggesting that PFKP may facilitate immune evasion through a glycolysis-immune axis. While existing studies have primarily focused on PFKFB3 and lactate metabolism in regulating the TME, reports on PFKP-mediated modulation of the TME remain extremely limited (26,27). Our results indicate that PFKP may be linked to elevated levels of immunoregulatory molecules and could be involved in a metabolism–signaling–immunity regulatory network in HCC. Tissue-level IHC analysis further supported a positive association between PFKP and PD-L1/CTLA-4 expression, consistent with a potential role for PFKP in shaping an immunoregulatory or potentially immunosuppressive microenvironment.

This study further found that patients with elevated PFKP expression exhibited higher IPS values, indicating a potentially greater predicted response to PD-1- and CTLA-4-targeted immune checkpoint blockade. Compared with conventional indicators such as PD-L1 expression alone or TMB, PFKP may provide additional information by reflecting both metabolic and immune-related features. These findings offer a potential perspective for immunotherapy stratification in HCC. Notably, PFKP not only reflects cellular metabolic status but also acts as a modulator of immune phenotypes, thereby participating in therapeutic response prediction. Further validation in prospective and treatment-annotated cohorts will be necessary to determine its clinical utility.

Drug sensitivity analysis showed that high PFKP expression was associated with lower predicted IC_50_ values for several antitumor agents, including paclitaxel, sunitinib, and quizartinib. These findings suggest that PFKP expression may be related to drug responsiveness and may have value as a candidate marker for therapeutic stratification. Unlike conventional approaches that mainly rely on genetic alterations or protein targets to estimate treatment response, our analysis incorporated metabolic features into the evaluation framework, highlighting the potential relevance of metabolic enzymes in precision oncology. Future studies are needed to determine whether integrating PFKP expression into treatment decision-making could help optimize drug selection, particularly in patients with limited therapeutic benefit.

The multifunctional profile of PFKP further supports its potential involvement in metabolism-immune crosstalk. Based on our findings, we propose a preliminary model in which PFKP upregulation is associated with enhanced glycolysis, activation of mTOR-related signaling, and increased PD-L1 expression, providing a possible framework for understanding how metabolic reprogramming may interact with immune regulation in HCC. This interpretation is consistent with emerging evidence that metabolic enzymes participate in oncogenic signaling and immune evasion networks (18,19). Our results help address the limited understanding of the immunoregulatory relevance of glycolysis in HCC and suggest that PFKP could be explored as a candidate target in combined metabolic-immune therapeutic strategies, particularly in view of the ongoing challenges in HCC management (20,21). A major strength of this study lies in its integrative design, which combines TCGA-based multi-omics analyses with validation in clinical specimens and *in vitro* experiments. Overall, these findings indicate that PFKP may have relevance not only for prognosis assessment but also as a candidate biomarker associated with both metabolic and immune features in HCC.

In summary, this study comprehensively elucidated the expression profile, mechanistic functions, and multidimensional value of PFKP in HCC, particularly in the contexts of immunotherapy prediction and drug sensitivity evaluation. Beyond its role as a rate-limiting enzyme in glycolysis, PFKP was associated with activation of mTOR-related signaling and increased expression of immune checkpoint molecules, suggesting a potential role in TME remodeling and immune regulation. These findings indicate that PFKP may have value as a candidate prognostic and therapeutic biomarker in HCC. By addressing the understudied intersection between metabolic enzymes and the TME, this study expands the current perspective on immunotherapy response prediction and suggests PFKP as a potential target for combined metabolic-immune intervention strategies. Several limitations should also be noted. First, the bioinformatics analyses were retrospective in nature, and the predictive value of PFKP for immunotherapy response requires further validation in prospective clinical cohorts. Second, the sample size of the local IHC validation cohort was relatively small, which may limit the generalizability of the findings. Third, although preliminary mechanistic investigations were performed *in vitro*, some evidence was primarily derived from cell line models and still requires validation in *in vivo* systems. Future studies incorporating PFKP knockout or knockdown models, pharmacological inhibition approaches, and immunotherapy-based animal models may help further clarify the biological role of PFKP and evaluate its translational relevance in HCC.

In summary, through integrated pan-cancer and HCC multicohort bioinformatics analyses, histological validation, and *in vitro* functional experiments, this study demonstrates that PFKP is markedly overexpressed in HCC and is closely associated with unfavorable OS, advanced T stage, and independent poor prognosis. Functional enrichment and GSEA analyses further suggest that PFKP-related genes are mainly involved in immune regulation and metabolic reprogramming. TME and immune profiling showed that elevated PFKP expression was accompanied by higher immune, stromal, and ESTIMATE scores, increased infiltration of activated dendritic cells, and upregulation of multiple immune checkpoint genes, while exhibiting a negative correlation with ADORA2A. In addition, IHC validation in clinical samples confirmed the increased expression of PFKP and Ki-67 and revealed positive correlations between PFKP and PD-L1 and CTLA-4. In HepG2 cells, PFKP overexpression promoted the expression of glycolysis-related enzymes HK2 and PKM2, enhanced PD-L1 and STAT3 expression, and activated the downstream mTOR signaling components p-S6K1 and p-4EBP1; these effects were partially reversed by 2-DG, supporting the existence of a potential functional axis of “PFKP → glycolysis → mTOR → immune factors.” Furthermore, IPS and drug sensitivity analyses suggested that patients with high PFKP expression may show greater responsiveness to PD-1/CTLA-4-based immunotherapy and several therapeutic agents, including quizartinib, paclitaxel, and sunitinib.

Overall, these findings deepen current understanding of the immune-metabolic coupling mechanisms in HCC and highlight PFKP as a potential biomarker and therapeutic target that links tumor glycolytic reprogramming with immune checkpoint regulation. Our study therefore provides new evidence for improving prognostic stratification, clarifying the molecular basis of metabolism–immunity crosstalk, and developing combined metabolic and immunotherapeutic strategies in HCC.

## CONFLICTS OF INTEREST

**Guarantor of the article:** Lilan Cen, PhD.

**Specific author contributions:** L.C.: Conceptualization, data curation, formal analysis, investigation, visualization, writing—original draft. Z.T.: Methodology, software, formal analysis, writing—review & editing. H.H.: Resources, investigation, project administration. S.L.: Visualization, validation, data curation. W.C.: Supervision, writing—review & editing, project administration. J.L.: Conceptualization, supervision, funding acquisition, writing—review & editing.

**Financial support:** This study was supported by the 2023 Self-funded research project of Health Commission of Autonomous Region (Grant No. Z-A20230026), 2024 Self-funded research project of Health Commission of Autonomous Region (Grant No. Z-A20240067), Baise scientific research and technology development project (Grant No. BaiKe 20233642).

**Potential competing interests:** The authors declare no conflict of interest.

**Availability of data and materials:** All data generated or analyzed during this study are included in this article and/or its supplementary material files. Further enquiries can be directed to the corresponding author.

**Study Highlights**

**WHAT IS KNOWN**

✓ Metabolic reprogramming, especially enhanced glycolysis, is a hallmark of hepatocellular carcinoma (HCC).
✓ The platelet-type phosphofructokinase (PFKP) is a key regulatory enzyme in glycolysis.
✓ The tumor immune microenvironment plays a crucial role in HCC progression and therapy resistance.

**WHAT IS NEW HERE**

✓ Integrated biomarker: PFKP overexpression is an independent prognostic factor and is positively correlated with the expression of immune checkpoint molecules (PD-L1, CTLA-4) in HCC tissues.
✓ Mechanistic link: PFKP functionally connects glycolytic flux to immune modulation by upregulating PD-L1 and activating the mTOR pathway, an effect dependent on its glycolytic activity.
✓ Therapeutic predictor: High PFKP expression is associated with a predicted favorable response to immune checkpoint inhibitors and specific chemotherapeutic agents, positioning it as a novel predictive biomarker for therapy selection.

## Supplementary Material

## ABBREVIATIONS:

2-DG: 2-Deoxy-d-Glucose
AUC: area under the curve
BP: biological process
CIs: confidence intervals
FDR: false discovery rate
GDSC: genomics of drug sensitivity in cancer
GO: gene ontology
GSEA: gene set enrichment analysis
HCC: hepatocellular carcinoma
HPA: human protein atlas
HR: hazard ratio
ICIs: immune checkpoint inhibitors
IHC: immunohistochemistry
IPS: immunophenoscore
KEGG: Kyoto Encyclopedia of Genes and Genomes
NES: normalized enrichment score
OS: overall survival
PFKP: platelet-type phosphofructokinase
ROC: receiver operating characteristic
TCGA: The Cancer Genome Atlas
TMB: tumor mutation burden
TME: tumor microenvironment
