# Supplementary material for: Platelet-Type Phosphofructokinase Drives Immune Checkpoint Coexpression and Metabolic Pathway Activation in Liver Cancer: The Cancer Genome Atlas–Based and Experimental Validation
Source: Clin Transl Gastroenterol. 2026 Apr 27;17(8):e01041. doi: 10.14309/ctg.0000000000001041 (PMC13502873; doi:10.14309/ctg.0000000000001041)
Supplement: Supplementary file 1 [file ct9-17-e01041-s001.docx]

**Table S1. Antibodies used for Western blot analysis.**

| **Target Protein** | **Manufacturer** | **Catalog Number** | **Dilution Ratio** | **Host Species** |
| --- | --- | --- | --- | --- |
| PFKP | Proteintech | 13389-1-AP | 1.430555556 | Rabbit |
| HK2 | Proteintech | 66974-1-IG | 3.513888889 | Mouse |
| PKM2 | Proteintech | 15822-1-AP | 1.430555556 | Rabbit |
| PD-L1 | Proteintech | 66248-1-IG | 0.736111111 | Mouse |
| STAT3 | Proteintech | 10253-2-AP | 0.736111111 | Rabbit |
| GAPDH | Proteintech | 60004-1-Ig | 0.736111111 | Mouse |
| p-S6K1 | Proteintech | 14485-1-AP | 1.430555556 | Rabbit |
| S6K1 | Abcam | ab167933 | 1.430555556 | Mouse |
| p-4EBP1 | Proteintech | 81812-4-RR | 0.736111111 | Rabbit |
| 4EBP1 | Abcam | ab32024 | 1.430555556 | Mouse |
